# Management of Anosmia in COVID-19: A Comprehensive Review

**DOI:** 10.7759/cureus.30425

**Published:** 2022-10-18

**Authors:** Khushi Khurana, Chandra Veer Singh

**Affiliations:** 1 Otolaryngology, Jawaharlal Nehru Medical College, Datta Meghe Institute of Medical Sciences, Wardha, IND; 2 Otolaryngology-Head and Neck Surgery, Jawaharlal Nehru Medical College, Datta Meghe Institute of Medical Sciences, Wardha, IND

**Keywords:** covid-19, treatment, management, anosmia, coronavirus, olfactory dysfunction, loss of smell

## Abstract

With the evolving understanding of COVID-19, a thorough analysis of the effects of this unique coronavirus on the affected people's olfactory abilities could highlight the disease's specific course of treatment. Researchers have discovered that the neurological side effects of SARS-CoV-2 infection include acute anosmia and ageusia. This work aims to review the relevant mechanisms, provide information on COVID-19-related anosmia, and suggest a novel approach to treating long-term anosmia brought on by coronavirus disease. For that, we did a thorough literature assessment of the subject from various online resources, including PubMed, Scopus, and Google Scholar. We evaluated the publications that described anosmia in COVID-19 and its management. In patients with SARS-CoV-2 infections, the angiotensin-converting enzyme two receptor plays a crucial role in the anosmia process. Olfactory systems are directly harmed by new coronaviruses when they connect with sustentacular cells' ACE-2 (Angiotensin converting enzyme-2) receptors. Other suggested processes include the virus's infiltration of the olfactory nerve and the ensuing local inflammation. Therefore, neuroprotective, anti-inflammatory, or depolarizing medications may be helpful for COVID-19 individuals who have lost their sense of smell. According to the available data, we found out olfactory training, topical or oral corticosteroids, caffeine, insulin, or minocycline may effectively treat COVID-19 odor loss. A novel method of treating long-term COVID-19 with persistent anosmia can be suggested based on recent investigations. The path to effective anosmia management is still somewhat hazy, but there is hope that we can find the right treatment plan with the right clinical trials and additional research. People who lost their sense of smell during COVID-19 can be reassured that recovery is typically possible.

## Introduction and background

SARS-COV-2 has been threatening millions of people since December 2019. The epidemic that is currently spreading across the globe is primarily caused by this new coronavirus [1]. SARS-CoV-2 belongs to the family coronaviridae. It is a single-stranded positive-sense RNA virus. This respiratory virus takes entry into the human cells via binding the angiotensin-converting enzyme 2 (ACE-2) receptors present in host cells. ACE-2 receptors are vastly illustrated in type 2 alveolar cells. Due to this high expression of ACE-2 receptors, the lungs become the most commonly affected site for developing the COVID-19 disease [2]. Acute anosmia and ageusia are found to be descriptive neurological complications of SARS-CoV-2 infection [3,4]. The majority of COVID-19 cases are reported to present with dry cough, fever, chills, myalgia, dyspnoea, sore throat, diarrhea, chest pain, sputum production, and nausea/vomiting [5,6].

Olfactory dysfunction is thought to affect 52.73% of COVID-19 patients, according to a meta-analysis by Tong et al. [4]. During the first 30 days of the disease, olfactory sensitivities were partially or completely restored in 63%-78% of anosmia related to COVID-19 patients. Even though the bulk of olfactory dysfunctions associated with COVID-19 appears to resolve quickly, some individuals have reported anosmia that lasted longer than 30 days [7]. Over 73% of patients had anosmia, which Kaye et al. recorded and showed had begun before their COVID-19 diagnosis. More than 26.6 percent of these people had anosmia as their initial symptom [8].

## Review

Methodology 

We carried out a thorough, comprehensive study of the literature using internet databases, including PubMed, Scopus, and Google Scholar. All feasible publications that described anosmia/symptoms/olfactory dysfunction in COVID-19 and management of COVID-19 were obtained. The keywords, "COVID-19", "coronavirus", "anosmia", "management", "treatment", "olfactory dysfunction", and "loss of smell" were used during the research. Alongside this, other data, such as the author's name, publication year, study design, and sample size, were taken into consideration in the current study. We have included 47 articles in this review.

Pathophysiology of anosmia

The expression of ACE-2 receptors on nasal mucosa may contribute to COVID-19 patient’s altered sense of smell [9]. Significant brain circuitry is dedicated to olfaction processing because the olfactory system offers crucial information from the environment. Respiratory and olfactory epithelium are the two types of epithelia that line the nasal cavity. The respiratory epithelium, which is a pseudo-stratified columnar epithelium comprised of ciliated cells, secreting cells or goblet cells, and also basal cells, covers the majority of the nasal cavity. The ciliated cells help to push mucus towards the distal aperture so that it can be expelled from the body, whereas the basal cells serve as progenitor cells that differentiate into the various cell types required for the respiratory epithelium. The goblet cells secrete mucus to moisten the epithelium [10]. Odor detection is the function of the olfactory epithelium. This epithelial layer contains duct cells of the olfactory (Bowman's) glands, basal cells, microvillar cells, sustentacular cells, and olfactory sensory neurons (OSNs) [11]. Odor information is carried to the brain from the olfactory epithelium via olfactory sensory neurons. Olfactory sensory neurons reach the olfactory bulb by passing through the cribriform plate at the base of the ethmoid bone of the skull. Reports suggest TMPRSS2 and ACE2 are the two main genes responsible for CoV-2 entry in the host cells. These are not expressed in the olfactory epithelium; instead, they are seen in supportive olfactory cells, stem cells, and perivascular cells [12], the sustentacular cells, or the supporting cells cushion the olfactory receptor neurons. Hence, any damage to the sustentacular cells can indirectly cause olfactory dysfunction [13]. This proposes that non-neuronal cells are involved in the changes in the perception of smell in COVID-19 [9,12,14].

Thirty-two patients who passed away from COVID-19 had their brain samples examined by Meinhardt et al. This finding provides evidence that the virus has an impact on olfactory sensory neurons. However, single immunocytochemical imaging cannot distinguish effectively betwixt neuronal cells and non-neuronal cells, especially in ancient samples which were collected shortly after death. Additionally, just three of the samples from the olfactory bulb contained viral ribonucleic acid, which did not clearly support the idea that the olfactory nerve was responsible for the virus's diffusion to the brain. Additionally, the analysis of the results is constrained by the absence of information regarding the patients who suffered anosmia [15]. Unlike the loss of smell sensation in other viral infections, anosmia in COVID-19 is not associated with inflammation of the nose, rhinorrhoea, or other coryzal symptoms [12,16]. Another study also implies that olfactory dysfunction may be linked with the sialic acid receptor and SARS-CoV-2 interaction; receptors are expressed on the nasal mucosa [17].

Localized Olfactory Epithelium Inflammation: Olfactory epithelial inflammation is one potential factor contributing to anosmia brought on by COVID-19. Upon contact with COVID-19 strains, olfactory epithelial cells reportedly expressed a high number of ACE2 receptors. In the olfactory epithelium, concentrations of local pro-inflammatory cytokines were examined by Torabi et al. They discovered that COVID-19 disease dramatically raised tumor necrosis factor-alpha levels. Anosmia linked to COVID-19 may have speedily renewed and recovered due to inflammation in the olfactory epithelium. It's possible that COVID-19 targets the nasal system cells that are present peripherally instead of the olfactory neurons because of the considerable expression of ACE-2 receptors in non-neuronal olfactory epithelial cells. Therefore, the olfactory can swiftly repair and recover well after a viral illness in situations where the virus damages the OE [12,18,19].

One of the studies suggests the direct invasion of the virus into the brain by anterograde axonal transport via olfactory sensory neurons [20]. Also, the reports from studies of various magnetic resonance imaging support the idea of changes in shapes and volumes of the olfactory bulb in cases of COVID-19 [21-23]. SARS-CoV-2 infiltrates both olfactory sensory neurons and sustentacular cells in men and models of Syrian hamsters exhibiting anosmia and ageusia related to COVID-19, as per the research by de Melo et al. published recently. This study focused on olfactory neuroepithelial cell death and identified mature olfactory sensory neuron apoptosis as the most significant contributor to anosmia in COVID-19 patients. It's noteworthy that even six months later the diagnosis of COVID-19, they discovered SARS-CoV-2 in the olfactory sensory neurons of patients with persistent anosmia. Although the results of this study validated the olfactory sensory neuron damage and potential neural invasion as causes of anosmia, additional research should specifically identify the malfunctioning of the olfactory bulb by utilizing bigger sample numbers and control groups [24].

Since rhinorrhea and nasal obstruction are very uncommon in COVID-19, they are excluded from the probable cause of olfactory dysfunction [25]. One of the prominent potential reasons for the development of hypo- or anosmia during viral infection can be an olfactory cleft blockage. Conductive loss results from odors not reaching the intact OE due to olfactory cleft occlusion, which also impacts airflow. In such situations, inflammation of nasal mucosa and its secretions in patients with viral illnesses leads to anosmia. However, a subset of COVID-19 individuals has been found to have acute onset anosmia without nasal congestion or discharge, suggesting a different mechanism for COVID-19 [21].

It is proposed that anosmia, ageusia, and headache are the presenting symptoms of milder cases. And these symptoms mark the inception of the disease [26]. Since these are very unspecific findings, it increases the chances of the disease being undiagnosed, leading to the spread of the disease [27]. Thus, discerning the management of anosmia in COVID-19 patients is becoming a current need.

Treatment

Presently, the potency of therapeutic options is not specified, but few treatments prove to be capable of solving the problem of olfactory dysfunction [28]. Olfactory training: It is persistent, and intended sniffing of odorants like eucalyptus oil, cloves, lemon, and rose symptomatically improves the olfactory dysfunction [29,30]. When begun early and with good compliance, olfactory training was reported to be most beneficial in enhancing olfactory function and was linked with fewer negative side effects [31].

As the use of corticosteroids is still open to question, various studies have put forth different results with the use of different corticosteroids [32,33].

Local mometasone furoate: A study by Abdelalim et al. was designed to know the effect of mometasone furoate on the improvement of anosmia in patients with COVID-19. The study was comprised of 100 confirmed COVID-19 cases, out of which 50 were taken to control. It demonstrates that the use of mometasone furoate provides no significant edge over olfactory training [32].

Nasal betamethasone drops: According to a randomized, double-blind placebo-controlled study, betamethasone has proved to have no noteworthy effect on the recovery time of anosmia in patients with COVID-19. Thus, as per this clinical trial, it is not recommended to use a nasal spray of betamethasone for the management of anosmia in COVID-19 [34]. 

Nasal fluticasone spray: Singh et al. evaluated the effect of fluticasone nasal spray and triamcinolone paste in 120 patients with complaints of loss of smell and loss of taste. The try-out smells in the experiment were peppermint, musky, pungent, floral, and camphoraceous. Out of 120 laboratory-controlled cases of SARS-CoV-2, 60 patients were considered as control, and the other 60 patients were taken as experimental cases. It was observed that the smell senses improved in the duration of a week tremendously in the cases as compared to the control. Accordingly, it can be expected that the use of fluticasone nasal spray can improve olfactory dysfunction in COVID-19 cases [35]. 

Coffee: A systematic review done by Hosseini A et al. has shown a remarkable result of caffeine in the revitalization of the sense of smell. The study also took patients with various underlying conditions into different groups. These conditions include diabetes, hypertension, and heart disease. The patients with no underlying conditions unveiled better and faster results with the consumption of caffeine in coffee. The effectuality of caffeine for underlying patients was between 2 days and 4 days, and that of outpatients was 5 to 7 hours [36,37]. 

Insulin: Lately, in 2021, Mohamad et al. developed some intranasal insulin films to test how well they treat anosmia brought on by the SARS-CoV-2 virus. Twenty of the 40 individuals who underwent randomization were given intranasal insulin films, while 20 were given a placebo. The insulin-treated group performed better on tests of odor discrimination and detection when the olfactory function of the two groups was compared. Additionally, analyzing scores before and after intervention revealed that insulin delivery, in contrast to the placebo group, led to considerably higher values post-intervention [38]. 

Minocycline: Minocycline has been shown to be effective in treating olfactory impairment. Minocycline could reduce olfactory sensory neuron apoptosis in rat models with bulbectomy, according to a histological study of animal olfactory tissue done by Kern et al. A normal sensory function reflects a balance between OSN apoptosis and regeneration. Consequently, employing the well-tolerated drug minocycline to suppress apoptosis may be justified in order to increase the quantity of olfactory sensory neurons [39]. 

Oral corticosteroids: A pilot trial done by Le Bon et al. The preliminary olfactory test was done on 72 patients who had been diagnosed with COVID-19 infection after about five weeks of loss of their sense of smell had disappeared. 37.5% of them displayed persistent dysosmia, and all were included in the study. Eighteen subjects underwent only olfactory training, while nine got oral corticosteroids and underwent olfactory training. Only those in the oral corticosteroid therapy and olfactory training group substantially raised their olfactory score. This result may indicate that a brief treatment of oral corticosteroids combined with olfactory training is both safe and potentially helpful for people with persistent dysosmia who have lost their sense of smell owing to COVID-19 [31]. In a case report by Touisserkani and Ayatollahi, 2020, on a 35-year-old lady who had recovered from COVID-19 and presented with anosmia, after a brief course of medication, oral prednisolone therapy had shown a good response, and the patient was treated. Thus, the study suggests when a patient's PCR swab test results come back negative, it can be advised to treat the anosmia with oral prednisolone [40].

Melatonin: Melatonin is a well-known anti-inflammatory, antioxidant, and immune-stimulating drug with an excellent safety record. Melatonin has a good cell diffusion capacity and high permeability into biological compartments, including the blood-brain barrier (BBB), because of its tiny size and amphiphilic characteristics. In order to prevent microvascular hyperpermeability and counteract SARS-CoV-2-induced neuroinvasion, melatonin restores BBB homeostasis. Additionally, melatonin's protective effects on olfactory sensory neurons have already been demonstrated in rat models. Furthermore, additional clinical information is required to investigate the effect of melatonin on the loss of smell and taste after COVID-19 [41,42].

Discussion

The mechanism of anosmia brought on by SARS-CoV-2 infection was evaluated in the current review, along with a few of the medications to cure them using pharmaceutical principles. In the COVID-19 era, this summary can be used to plan new clinical studies. SARS-CoV-2-induced anosmia has several significant characteristics. First, a sizeable percentage of patients with SARS-CoV-2 have these symptoms, which may be the mere characteristics of the illness. Additionally, these symptoms typically appear all of a sudden and last only a short while. Furthermore, they typically have nothing to do with nasal congestion [27,43]. Although not life-threatening, these symptoms have a negative impact on quality of life and are linked to sadness, anxiety, and an increase in suicidal thoughts [44,45].

Although the exact pathophysiology of anosmia is unknown, numerous research point to a number of potential causes. The most plausible causes of the SARS-CoV-2-induced anosmia among the proposed processes are local inflammation and direct damage to the sustentacular cells. Neuronal damage, including direct injury to olfactory receptor neurons, was previously thought to be the least likely cause for two causes: one, ACE2 and TMPRSS2 are not manifested in olfactory receptor neurons; and second, symptomatic improvement typically takes place faster than olfactory receptor neurons regeneration. Nonetheless, nasal samples and the results of MRI (magnetic resonance imaging) revealed that neuronal infection along with central nervous system invasion is a crucial factor in anosmia due to COVID-19. Particular consideration should be given to the neuronal damage in COVID-19 patients who have persistent anosmia [21-24,46].

Several drugs have been recommended in combination to treat anosmia. Olfactory training has been proposed in the past as a reliable and effective remedy for olfactory impairment. To cure olfactory impairment, however, there is no approved medicine. Corticosteroids have been studied the most out of all the drugs mentioned in COVID-19. The use of systemic corticosteroids for SARS-CoV-2-mediated olfactory dysfunctions, however, should be recognized as it may carry extra hazards and may slow the body's ability to rid itself of the virus [31,40,47].

## Conclusions

In accordance with the most recent literature studies, a sizable portion of COVID-19 patients has anosmia symptoms, for which the cellular causes and molecular causes are yet to be known. Since the olfactory epithelium exhibits a markedly strong expression of ACE2 receptors, inflammation in this region may be one of the primary causes of anosmia. Despite the absence of ACE2 receptors in the olfactory receptor neurons, inflammation may spread to olfactory receptor neurons or stem cells via the supporting cells and may harm the olfactory bulb as well as the central nervous system, leading to loss of smell sensation. Most coronavirus disease-related anosmia cases recover quickly, and the most important contributing factors to this anosmia are probably injury to the olfactory epithelial and inflammation of olfactory cilia or alterations in odor transmission. Olfactory training, intranasal mometasone furoate, nasal fluticasone, local betamethasone, caffeine, minocycline, or oral corticosteroids are all possible treatments. Anosmia should be added to the list of symptoms used in screening tools for potential COVID-19 infection, according to the proposed investigation and the continuously emerging body of evidence, even though more research is still needed. It is no longer acceptable for public health organizations to ignore this symptom. We can reassure folks who have lost their sense of smell during COVID-19 that recovery is generally achievable.
